# Bioinformatic Profiling Identifies a Fatty Acid Metabolism-Related Gene Risk Signature for Malignancy, Prognosis, and Immune Phenotype of Glioma

**DOI:** 10.1155/2019/3917040

**Published:** 2019-12-04

**Authors:** Ying Qi, Di Chen, Qiqi Lu, Yu Yao, Chunxia Ji

**Affiliations:** ^1^Department of Neurosurgery, Huashan Hospital, Fudan University, Shanghai 200040, China; ^2^Immunology Laboratory, Neurosurgical Institute of Fudan University, Shanghai 200040, China

## Abstract

Cancer cells commonly have metabolic abnormalities. Aside from altered glucose and amino acid metabolism, cancers cells often share the attribute of fatty acid metabolic alterations. However, fatty acid metabolism related-gene set has not been systematically investigated in gliomas. Here, we provide a bioinformatic profiling of the fatty acid catabolic metabolism-related gene risk signature for the malignancy, prognosis and immune phenotype of glioma. In this study, a cohort of 325 patients with whole genome RNA-seq expression data from the Chinese Glioma Genome Atlas (CGGA) dataset was used as training set, while another cohort of 667 patients from The Cancer Genome Atlas (TCGA) dataset was used as validating set. After confirmed that fatty acid catabolic metabolism-related gene set could distinguish clinicopathological features of gliomas, we used LASSO regression analysis to develop a fatty-acid metabolism-related gene risk signature for glioma. This 8-gene risk signature was found to be a good predictor of clinical and molecular features involved in the malignancy of gliomas. We also identified that this 8-gene risk signature had high prognostic values in patients with gliomas. Correlation analysis showed that our risk signature was closely associated with the immune cells involved in the microenvironment of glioma. Furthermore, the fatty acid catabolic metabolism-related gene risk signature was also found to be significantly correlated with immune checkpoint members *B7-H3* and *Tim-3*. In summary, we have identified a fatty acid metabolism-related gene risk signature for malignancy, prognosis, and immune phenotype of glioma; and our study might contribute to better understanding of metabolic pathways and further developing of novel therapeutic approaches for gliomas.

## 1. Introduction

Abnormality of metabolism is the hallmark of cancer, and identification of the metabolic weaknesses of cancer cells has prompted new therapeutic approaches toward tumor treatments [1]. For example, increased glucose metabolism has been frequently seen as a characteristic of cancer cells [2]. Except for glucose metabolism, altered fatty acid metabolism in cancer cells has received increasing attention recently [3]. Fatty acid is the cornerstone of cell membrane formation, energy storage, and signaling molecule production in carcinogenesis; thus targeting at the pathway of fatty acid metabolism might inhibit rapid proliferation of the cancer cells [4].

In this study, we focus on one of the fatty acid metabolism-related gene sets – the catabolic metabolism of fatty acid gene set in gliomas. Gliomas are the most prevalent and malignant primary brain tumors in adults, and glioblastoma (GBM) is the most common and devastating type among all grades of glioma. Despite of the novel therapy of GBM, patients with GBM only have a median overall survival time of 14.6-16.7 months in clinical trials [5]. Metabolic profiling analysis of gliomas might contribute to better understanding of molecular pathways and further developing of novel therapies in gliomas [6]. For example, bioinformatic profiling had demonstrated a glucose metabolism-related risk signature and an amino acid metabolism-related risk signature were closely associated with malignancy and prognosis of glioma [7, 8]. Through mass spectrometry, lipidomic signatures were also found to be approaches for classification of gliomas [9]. However, the role of the fatty acid metabolism-related gene set in glioma still remains unclear.

In our study, we firstly identified that the fatty acid catabolic metabolism-related gene set had the ability to distinguish clinicopathological features of gliomas. Then, we generated a fatty-acid catabolic metabolism-related gene risk signature in CGGA dataset, and further validated in TCGA dataset. We observed that our risk signature was associated with molecular features of gliomas and could serve as an independent prognostic factor for both all grade gliomas and GBM. Lastly, we also found that this risk signature was closely related to tumor infiltrating lymphocytes, which indicated an potential association between fatty acid metabolism and immune phenotype of gliomas. We believe that our results might provide a new insight for understanding the metabolic mechanism of gliomas.

## 2. Methods and Materials

### 2.1. Data Collection

The whole genome RNA-seq expression data and clinical information of 325 glioma patients from CGGA dataset (http://www.cgga.org.cn) were used as the training set [7, 8]. RNA-seq data and clinical information from TCGA dataset (http://cancergenome.nih.gov) were used as validation set [10, 11]. After dropping the samples with severely incomplete data (e.g. lack of critical clinical information such as overall survival time and IDH status), the eventual size of validation set was 667.

### 2.2. Bioinformatics Analysis

Fatty acid catabolic metabolism-related gene set (GO_FATTY_ACID_CATABOLIC_PROCESS), consisted of 73 genes in total, was extracted from Molecular Signatures Database v6.2 (http://www.broad.mit.edu/gsea/msigdb/) [7]. Most variable genes of the gene set, determined by their median absolute deviation (MAD), were selected for further consensus clustering [12]. Consensus clustering was carried out in R programming language (http://cran.r-project.org) for detecting the fatty acid catabolic metabolism-related glioma subgroups of the training set. The optimal number of the glioma clusters was determined by quantitative stability evidence in an unsupervised analysis. For evaluating the correlation between risk signature and the immune phenotype of glioma, the ESTIMATE package of R programming language was conducted to calculate the immune score which represented the infiltration of immune cell in the microenvironment of glioma [13]. The association between immune cells and glioma risk signature was analyzed by Gene Set Variation Analysis (GSVA) in R programming language as described by Zhang and colleagues [14, 15].

### 2.3. Statistical Analysis

Screened by univariate Cox regression analysis in the training set, 46 genes with prognostic significance (p < 0.05) in fatty acid catabolic metabolism-related gene set were selected for Least Absolute Shrinkage and Selection Operator (LASSO) regression analysis [16, 17]. The generalized linear model produced by LASSO regression analysis was further analyzed with 10-fold cross validation in order to generate the minimum cross validated error. Based on the cross validation, 8 genes with their regression coefficients (Coef) were eventually achieved. Then the risk score for each patient in the training set and validation set was calculated by the following formula:

(1) $$\begin{array}{c} Risk\cdot score={\text{expr}}_{\text{gene}1}\times {Coef}_{\text{gene}1}+{\text{expr}}_{\text{gene}2}\times {Coef}_{\text{gene}2}+\cdots +{\text{expr}}_{\text{gene}8}\times {Coef}_{\text{gene}8} \end{array}$$

All patients in the training set and validation set were then separated into high or low risk group according to the median risk score. Survival analysis based on the risk score was evaluated by Kaplan-Meier survival curve by using R programming language. Univariate and multivariate survival analysis was performed by using Cox proportional hazards model in R programming language. Other main statistical analysis including Student's t-test, chi-square test, and Pearson's test were all performed in R programming language. Statistical significance was considered at the level of *p* < 0.05.

## 3. Results

### 3.1. Classification of Gliomas Based on Fatty Acid Catabolic Metabolism-Related Gene Set

**Figure 1:**
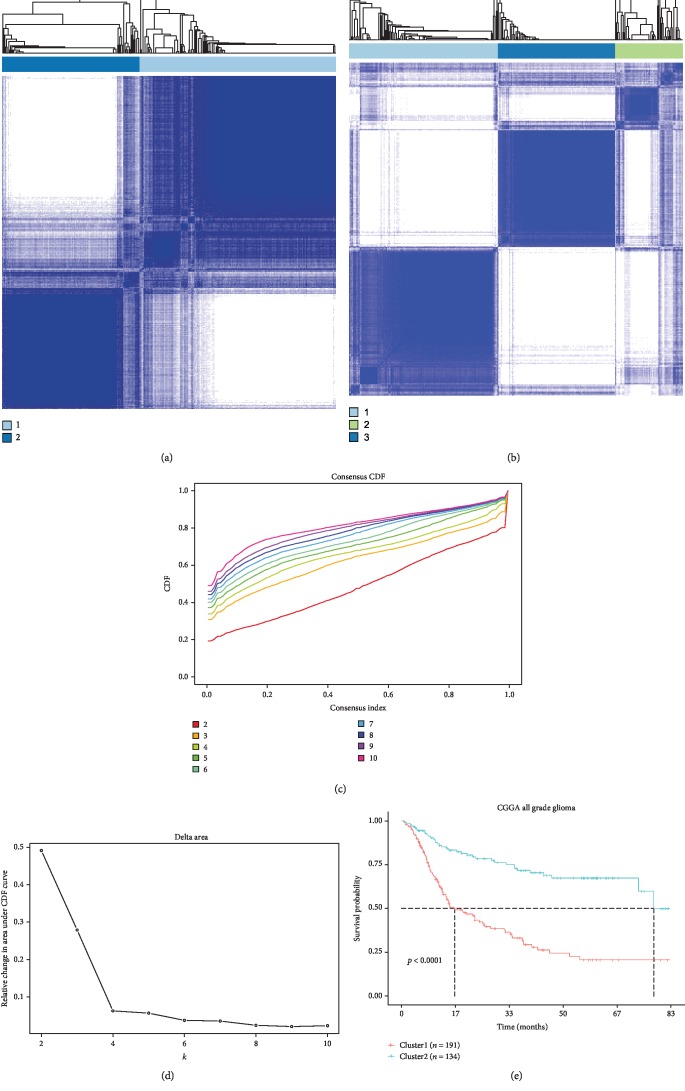
Classification of gliomas based on fatty acid catabolic metabolism-related gene set in CGGA dataset. (a) and (b) Consensus clustering matrix of 325 CGGA samples for *k* = 2 and *k* = 3. (c) Consensus clustering CDF for *k* = 2 to *k* = 10. (d) Relative change in area under CDF curve for *k* = 2 to *k* = 10. (e) Kaplan-Meier survival analysis of two clusters classified by consensus clustering in the training set.

The gene expression profiling of the 73 fatty acid catabolic metabolism genes obtained from the training set was used as variables of consensus clustering. The result of consensus clustering indicated that 325 patients in the training set could be classified into two robust clusters with clustering stability increasing between *k* = 2 to *k* = 10 (Figures 1(a)-1(d), [Supplementary-material supplementary-material-1]). Kaplan-Meier survival analysis showed that patients with gliomas in cluster1 had a significantly poorer prognosis than in cluster2 (Figure 1(e)). Furthermore, differences in clinicopathological features between these two clusters were also found. Cluster1 had a strong correlation with older age at diagnosis (median age: 45, *p* < 0.001), classical or mesenchymal subtypes (66.85%, *p* < 0.001), glioblastoma phenotype (63.54%, *p* < 0.001), IDH wildtype (75.14%, *p* < 0.001), and 1p/19q non-codeletion (87.29%, *p* < 0.001; [Supplementary-material supplementary-material-1]). By contrast, cluster2 mainly represented younger age at diagnosis (median age: 39, *p* < 0.001), proneural or neural subtypes (86.92%, *p* < 0.001), lower grade phenotype (80.77%, *p* < 0.001), IDH mutation (86.15%, *p* < 0.001), and 1p/19q non-codeletion (68.66%, *p* < 0.001, [Supplementary-material supplementary-material-1]). Our results indicated that fatty acid catabolic metabolism-related gene set was involved in the malignancy of gliomas and strongly correlated to prognosis.

### 3.2. Identification of an 8-Gene Risk Signature Associated with Fatty Acid Catabolic Metabolism

**Figure 2:**
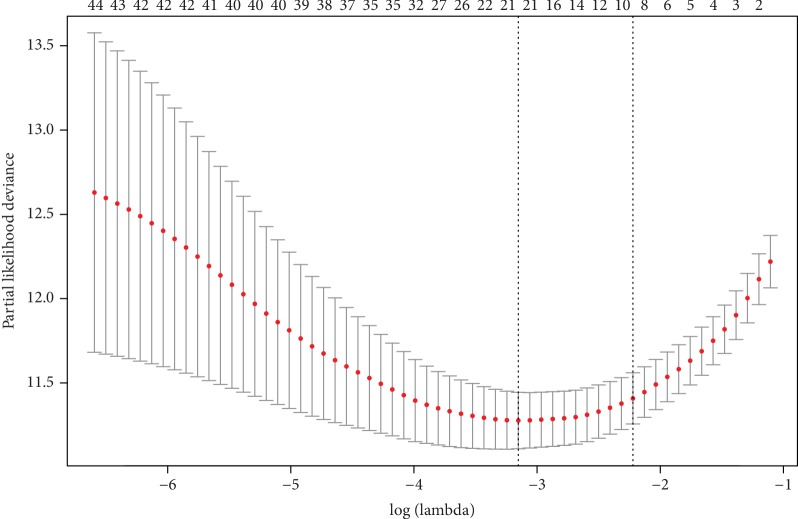
LASSO regression analysis of all genes with high prognostic values generated 8 genes as active covariates to evaluate the prognostic value. Red points represent log(lambda) value and gray bars represent confidence intervals of the cross-validated error. Top horizontal numbers represent number of all the genes involved in each Lasso regression fitting methods. Left vertical dotted line represents the log(lambda) value with minimum error, whereas the right vertical dotted line represents the largest log(lambda) value with 1SD of the minimum error.

Through univariate Cox regression analysis, 46 fatty acid catabolic metabolism-related genes with prognostic significance (*p* < 0.05) were selected for further analysis in the training set. To identify the gene risk signature associated with fatty acid catabolic metabolism, these 46 genes were undergone the LASSO regression analysis. After cross validation, LASSO regression analysis generated 8 genes (*ABCD1, ACADSB, CEL, CPT2, GCDH, NUDT19, PCCA, PEX13*) in total as active covariates to calculate the risk score (Figure 2 and Table 1). The signature risk score of each patient in the training set and validating set was then calculated with the LASSO regression coefficients and expression value of these 8 genes through formula (1) mentioned above.

### 3.3. 8-Gene Risk Signature Distinguished the Clinicopathological Features of Gliomas

**Figure 3:**
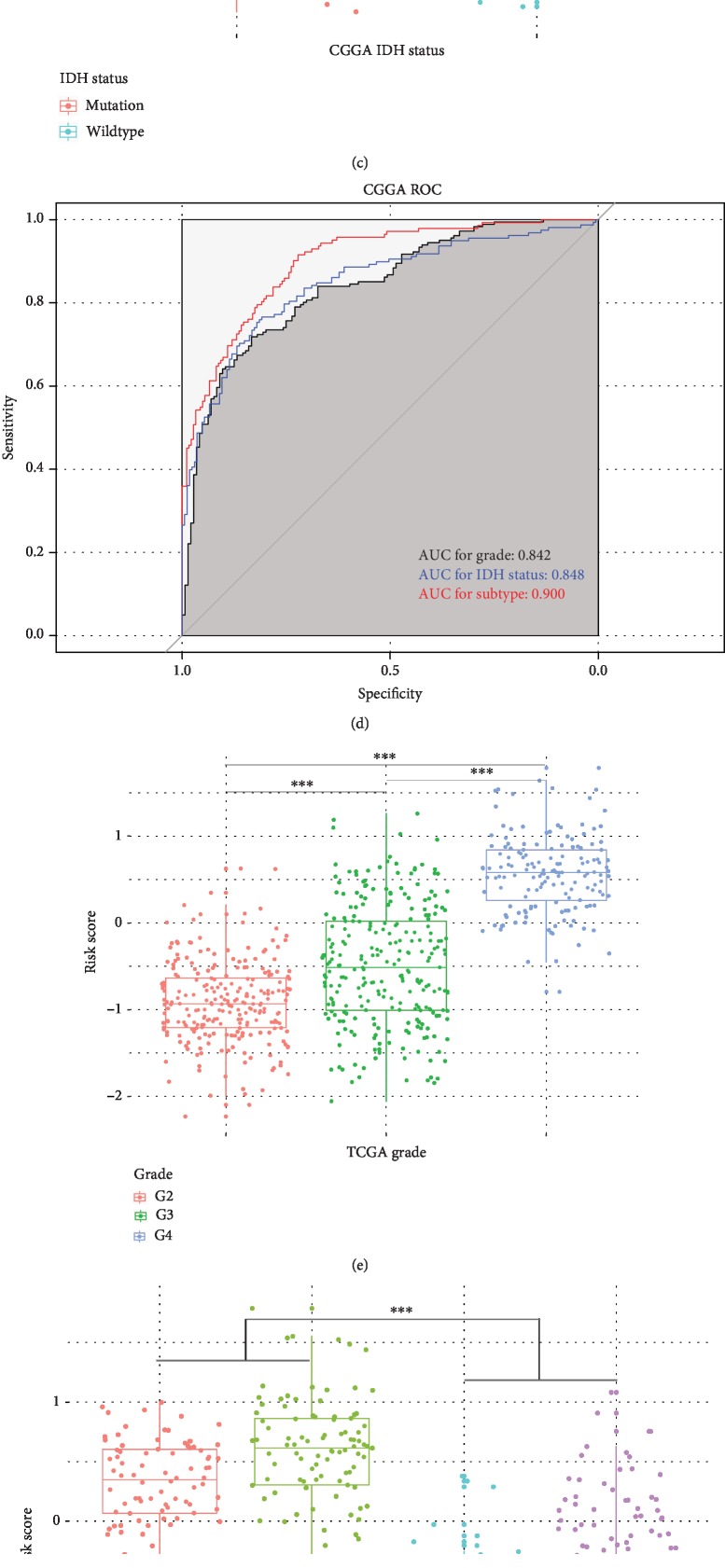
8-gene risk signature distinguished the clinicopathological features of gliomas. (a)-(c) Distribution of the 8-gene risk signature in CGGA patients with different grades, subtypes and IDH status. (d) ROC curves of grade, IDH status, and subtype with risk signature in the CGGA datasets. (e)-(g) Distribution of the 8-gene risk signature in TCGA patients with different grades, subtypes and IDH status. (h) ROC curves of grade, IDH status, and subtype with risk signature in the TCGA datasets (^∗∗∗^*p* < 0.05).ROC, receiver operating characteristic; AUC, area under curve; IDH, isocitrate dehydrogenase.

After calculating 8-gene risk signature score of each patients, we observed that higher risk scores were found in glioblastoma than lower grade gliomas (*p* < 0.001), in classical and mesenchymal subtypes than other subtypes (*p* < 0.001), in IDH wildtype than IDH mutation (*p* < 0.001) in the CGGA dataset (Figures 3(a)-3(c)). Similar distributional pattern of the risk score was also observed in TCGA dataset (Figures 3(e)-3(g)). Receiver operating characteristic (ROC) curves in both CGGA and TCGA datasets showed risk signature could serve as a good predictor for grade, IDH status and molecular subtypes of gliomas (Figures 3(d)-3(h)). Then, we classified the patients in training set into high risk group and low risk group by using median signature risk score as the cutoff value. Patients in high risk groups were linked to older age at diagnosis (median age: 47.5, *p* < 0.001), classical or mesenchymal subtypes (73.45%, *p* < 0.001), glioblastoma phenotype (70.37%, *p* < 0.001), IDH wildtype (75.31%, *p* < 0.001), and 1p/19q non-codeletion (93.96%, *p* < 0.001, [Supplementary-material supplementary-material-1]). By contrast, patients in low risk groups were associated with younger age at diagnosis (median age: 39, *p* < 0.001), proneural or neural subtypes (85.89%, *p* < 0.001), lower grade phenotype (81.60%, *p* < 0.001), IDH mutation (77.91%, *p* < 0.001) and 1p/19q non-codeletion (70.51%, *p* < 0.001, [Supplementary-material supplementary-material-1]). In TCGA dataset, we also observed that patients in high risk group were correlated with older age at diagnosis (median age: 54, *p* < 0.001), classical or mesenchymal subtypes (65.19%, *p* < 0.001), IDH wildtype (64.74%, *p* < 0.001), and 1p/19q non-codeletion (96.32%, *p* < 0.001, [Supplementary-material supplementary-material-1]); while patients in low risk group had a strong correlation with younger age at diagnosis (median age: 40, *p* < 0.001), proneural or neural subtypes (97.31%, *p* < 0.001), lower grade phenotype (99.70%, *p* < 0.001), IDH mutation (93.36%, *p* < 0.001) and 1p/19q non-codeletion (53.29%, *p* < 0.001, [Supplementary-material supplementary-material-1]). These results indicated that the 8-gene risk signature associated with fatty acid catabolic metabolism could distinguish the malignancy of gliomas.

### 3.4. Prognostic Value of 8-Gene Risk Signature in All Grade Gliomas and Glioblastoma

**Figure 4:**
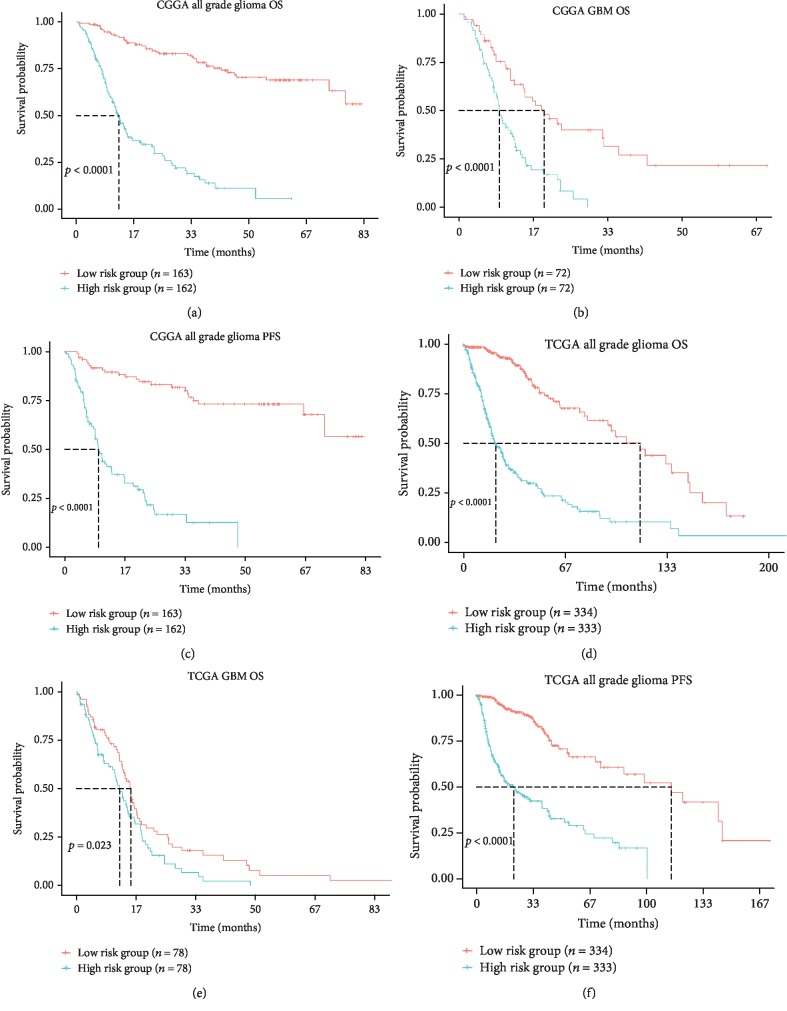
Prognostic value of 8-gene risk signature in CGGA and TCGA dataset. (a)-(c) Kaplan-Meier Survival curves of the 8-gene risk signature for all grade gliomas and GBM in CGGA dataset. (d)-(f) Kaplan-Meier Survival curves of the 8-gene risk signature for all grade gliomas and GBM in TCGA dataset. OS, overall survival; PFS, progression-free survival; GBM, glioblastoma.

In the CGGA dataset, Kaplan-Meier survival analysis revealed that patients in high risk group (*n* = 162) had a significantly poorer prognosis compared with patients in low risk group (*n* = 163; median OS: 10.5 vs 37.1 months; *p* < 0.001; Figure 4(a)). In TCGA dataset, patients in high risk group (*n* = 333) were also found to have much shorter overall survival times than patients in low risk group (*n* = 334, median OS: 15.5 vs 24.3 months; *p* < 0.001; Figure 4(d)). After taking important clinical and molecular factors (including age, gender, WHO grade, IDH status, chemotherapy and radiotherapy) into account, univariate and multivariate Cox analysis further demonstrated that this risk score was an independent prognostic factor of prognosis in CGGA dataset (Table 2). Cox proportional hazard model also found risk score could serve as an independent prognostic factor in TCGA dataset (Table 2). When focusing on the GBM phenotype, we also observed that patients in high risk group (*n* = 72) had a shorter OS than patients in low risk group (*n* = 72) of GBM phenotype in CGGA dataset(median OS: 8.5 vs 11.5 months; *p* < 0.001; Figure 4(b)). Results in TCGA dataset further validated the prognostic value of the risk signature in GBM phenotype (Figure 4(e)). In addition, the progression-free survival time of the high risk group was much shorter than low risk group in both CGGA and TCGA datasets (Figures 4(c)-4(f)). These results indicated that our 8-gene risk signature associated with fatty acid catabolic metabolism had high prognostic value in both all grade gliomas and glioblastoma.

### 3.5. Correlation of the Gene Signature and Immune Phenotype of the Gliomas

**Figure 5:**
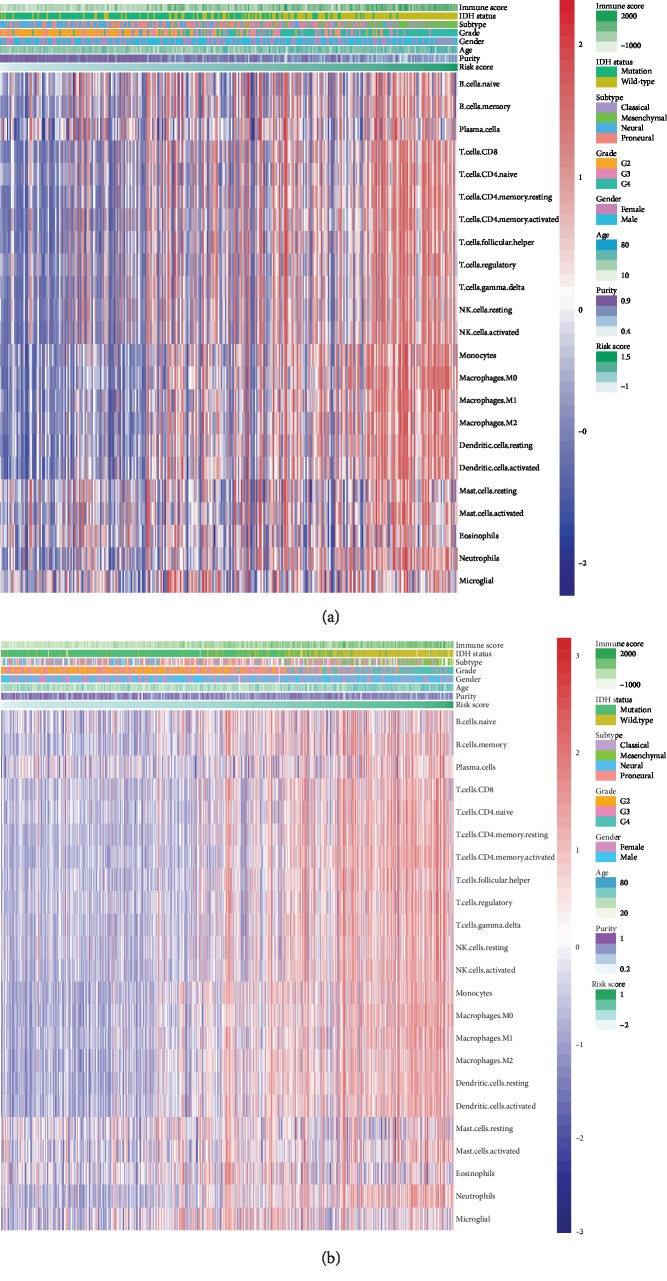
Correlation of the 8-gene risk signature and immune microenvironment of the gliomas. (a) Heat map shows the association of risk scores and immune cells in CGGA datasets. (b) Heat map shows the association of risk scores and immune cells in TCGA datasets.

To investigate the role of 8-gene risk signature in the immune phenotype of gliomas, we used GSVA method to calculate the immune score and immune cell populations of glioma samples in the CGGA dataset and TCGA dataset. Through Pearson correlation test, the gene signature was found to be closely associated with immune score (*R* = 0.624), activated CD4^+^ T cells (*R* = 0.501), monocytes (*R* = 0.545), macrophages (*R* = 0.621), and activated NK cells in CGGA dataset (*R* = 0.490, Figure 5(a)). Similarly, we observed the risk score had a strong correlation with immune score (*R* = 0.640), activated CD4^+^ T cells (*R* = 0.678), monocytes (*R* = 0.583), macrophages (*R* = 0.651), and activated NK cells in TCGA dataset (*R* = 0.581, Figure 5(b)). Furthermore, our gene signature also showed a moderate correlation with CD8^+^ T cells in both datasets(CGGA, *R* = 0.455; TCGA, *R* = 0.492). In addition, the 8-gene risk signature was found to be positively correlated with immune checkpoints related molecules including *CD274*, *CD276*, *HAVCR2*, *LAG3*, and *PDCD1* through Pearson correlation test ([Supplementary-material supplementary-material-1]-[Supplementary-material supplementary-material-1]). Among them, *CD276* (also known as *B7-H3*; CGGA, *R* = 0.56; TCGA, *R* = 0.72) and *HAVCR2* (also known as *Tim-3*; CGGA, *R* = 0.47; TCGA, *R* = 0.53) had a significant correlation with 8-gene risk signature. These results indicated that the 8-gene risk signature associated with fatty acid catabolic metabolism might have a strong correlation with altered immune microenvironment of the gliomas.

## 4. Discussion

Altered cancer metabolic processes such as glucose metabolism and amino acid metabolism are the hallmarks of cancers [18]. Metabolomic signatures can provide a better understanding of the molecular pathways of gliomas and offer great potentials for developing novel therapeutic approaches in glioma treatments [6]. For example, metabolic pathways including cysteine metabolism, nucleotides metabolism and 2-hydroxyglutarate have been demonstrated to be helpful for classification of gliomas [19, 20]. Myo-inositol, an important osmolyte and substrate in phosphatidylinositol lipid family, was also found to be associated with glioma grade [21]. Previous studies have also identified an amino acid metabolism-related gene risk signature and a glucose metabolism-related gene risk signature both have high prognostic value in glioma through bioinformatic analysis [7, 8]. Nevertheless, the role of the fatty acid metabolism-related gene set in glioma still remains unclear.

To date, this is the first study introducing a fatty acid catabolic metabolism-related gene risk signature for the malignancy of gliomas and the survival of patients with gliomas. After confirmed that 73 fatty acid catabolic metabolism-related genes had the ability to distinguish the key clinicopathological features of gliomas in both CGGA and TCGA datasets, we built a fatty acid catabolic metabolism-related gene risk signature through LASSO regression analysis. Lower grade gliomas (LGG, WHO grade II and III) have a preferentially better clinical and prognostic characteristics compared with higher grade gliomas (HGG, WHO grade IV) [22]. IDH status and TCGA molecular subtypes(classical, mesenchymal proneural and neural) were key features for classification and prognosis of glioma [11, 23]. Using our risk signature, patients in higher risk group tend to be associated with the higher grade, the more invasive TCGA molecular subtypes (classical and mesenchymal) and IDH wide type, which represented worse prognosis. By contrast, the lower grade, the less invasive TCGA subtypes (proneural and neural subtype) and IDH mutation were preferentially associated with patients in lower risk group. Furthermore, several oncometabolites have been confirmed to be the accumulated metabolic products of IDH mutation. For example, the abnormal glucose metabolite 2-hydroxyglutarate was a metabolic biomarker in gliomas and was useful in classification of gliomas [24]. However, the association between fatty acid metabolite and IDH mutation still remains unclear and our risk signature might provide some clues in this researching field. In summary, our results indicated that fatty acid catabolic metabolism might be involved in the progression of gliomas.

To further explored the potential role of fatty acid catabolic metabolism in gliomas, we evaluated the correlations between our risk signature and immune cell populations. Previous studies have demonstrated that tumor infiltrating lymphocytes(TILs), especially the CD4^+^ T cells and CD8^+^ T cells, are correlated with clinical prognosis in gliomas [25, 26]. Our study found fatty acid metabolic gene risk signature was highly associated with CD4^+^ T cells and moderately correlated with CD8^+^ T cells, which indicated that patients of higher risk tend to have an unfavorable TILs pattern as previously demonstrated: lower level of CD8^+^ T cells combined with higher level CD4^+^ T cells [26]. In addition, our risk signature was also strongly correlated with innate immune cells including monocytes, macrophages, and NK cells, which might be consistent with previous studies that tryptophan metabolic adaptations in GBM were associated with evasion of innate immune system by tumors cells [27]. Our findings indicated that fatty acid catabolic metabolism-related gene risk signature might, to some extent, involve in the altered immune microenvironment of the gliomas. Furthermore, we evaluated the correlation of our gene risk signature and common immune checkpoint members. *B7-H3* and *Tim-3*, the novel targets of immunotherapy against solid tumors [28, 29], both showed close association with risk signature. Clinical trials of anti-B7-H3 (NCT02475213) and anti-Tim-3 (NCT02817633) are carrying on, and our study showed the fatty acid catabolic metabolism-related gene risk signature might be a possible metabolic marker of the immunotherapy for gliomas.

In our fatty acid catabolic metabolism-related signature, the protein encoded by *ABCD1* is one of the superfamily of ATP-binding cassette transporters and is involved in the catabolic metabolism of very long chain fatty acid. *ABCD1* is associated with altered white matter microvascular perfusion [30] and may contribute to the cell differentiation with parallel to tumorigenesis [31]. In previous study, *ABCD1* transcript levels were overexpressed in breast cancer [32]. The protein encoded by *CPT2* is a nuclear protein transported to the mitochondrial membrane. *CPT2* plays a critical role in regulation of fatty acid oxidation [33] and might promote carcinogenesis in liver cancer by leading hepatocellular carcinoma to lipid-rich environment [34]. *PCCA*, encoding the mitochondrial enzyme Propionyl-CoA carboxylase, was also found to be altered in gastric and colorectal cancer [35]. Relationship of gliomas and other proteins encoded by the genes of our risk signature remains unclear and needs further researches. In summary, our fatty acid metabolic gene risk signature model may provide new insights into the carcinogenesis and therapeutic approaches of gliomas.

## 5. Conclusion

In conclusion, we identified a fatty acid catabolic metabolism-related gene risk signature for the malignancy, prognosis and immune phenotype of gliomas, and our study might provide better understanding of fatty acid metabolic role in glioma carcinogenesis and in glioma immune phenotype.

## Figures and Tables

**Table 1:** Univariate Cox regression analysis and LASSO regression coefficients of 8 genes generated by LASSO regression analysis.

Gene	HR	95% CI	*p* value	LASSO regression coefficient
ABCD1	1.422	1.323~1.528	<0.0001	0.09507427
ACADSB	0.6162	0.5537~0.6857	<0.0001	-0.16186716
CEL	1.476	1.275~1.709	<0.0001	0.06987048
CPT2	1.304	1.208~1.408	<0.0001	0.04503572
GCDH	0.7325	0.6504~0.825	<0.0001	-0.03428093
NUDT19	1.431	1.327~1.543	<0.0001	0.03613675
PCCA	0.5153	0.4348~0.6107	<0.0001	-0.12505459
PEX13	1.675	1.468~1.911	<0.0001	0.13984067

CI, confidence interval; HR, hazard ratio.

**Table 2:** Univariate and multivariate Cox regression analysis of the clinical features and risk score for overall survival in CGGA and TCGA datasets.

	Univariate analysis			Multivariate analysis		
Variables	HR	95% CI	*p* value	HR	95% CI	*p* value
Training set CGGA RNA-seq cohort (*n* = 325)						
Age	1.038	1.023~1.054	<0.0001	0.9992	0.9832~1.0155	0.9239
Gender	1.1701	0.8291~1.651	0.371	1.2047	0.8257~1.7576	0.3340
Grade	0.1697	0.1167~0.2469	<0.0001	0.5155	0.3203~0.8295	0.0063
IDH status	4.285	2.971~6.181	<0.0001	0.9195	0.5404~1.5643	0.7568
Chemotherapy	1.233	0.8736~1.74	0.234	0.9005	0.6223~1.3030	0.5782
Radiotherapy	0.4056	0.2839~0.5795	<0.0001	0.3655	0.2471~0.5406	<0.0001
Risk score	5.118	3.913~6.695	<0.0001	4.0044	2.7634~5.8028	<0.0001
Validation set TCGA RNA-seq cohort (*n* = 667)						
Age	1.067	1.057~1.077	<0.0001	1.0283	1.0142~1.0425	<0.0001
Gender	1.236	0.9566~1.596	0.105	1.3371	0.9729~1.8377	0.0734
Grade	0.11	0.08377~0.1444	<0.0001	0.6047	0.4021~0.9095	0.0157
IDH status	9.775	7.365~12.97	<0.0001	2.7861	1.7093~4.5413	<0.0001
Chemotherapy	0.4079	0.274~0.6073	<0.0001	0.6478	0.4238~0.9903	0.0391
Radiotherapy	2.121	1.532~2.937	<0.0001	0.9892	0.5870~1.6670	0.9675
Risk score	4.172	3.503~4.969	<0.0001	1.7382	1.0577~2.8567	0.0292

Gender (female and male); Grade (WHO grade IV and III, II); IDH status (wildtype and mutant); Risk score (low and high); Chemotherapy (treated and untreated); Radiotherapy (treated and untreated). CI, confidence interval; HR, hazard ratio; IDH, isocitrate dehydrogenase.

## Data Availability

All the data websites were confirmed to be available online.

## Supplementary Materials

Supplementary Materials Figure S1 Consensus matrixes for *k* = 4 to *k* = 10 of the 325 patients in the CGGA datasets by clustering the gene expression profile of the 73 fatty acid metabolism genes. Figure S2: Correlation of 8-gene risk signature and immune checkpoints related molecules in CGGA and TCGA datasets. Table S1: Clinicopathological features of two clusters classified by consensus clustering based on fatty acid catabolic metabolism-related gene set in CGGA dataset. Table S2: Clinicopathological features of two gene risk group in CGGA and TCGA dataset.

## References

[1] Schulze A., Harris A. L. (2012). How cancer metabolism is tuned for proliferation and vulnerable to disruption. *Nature*.

[2] Gatenby R. A., Gillies R. J. (2004). Why do cancers have high aerobic glycolysis?. *Nature Reviews Cancer*.

[3] Carracedo A., Cantley L. C., Pandolfi P. P. (2013). Cancer metabolism: fatty acid oxidation in the limelight. *Nature Reviews Cancer*.

[4] Currie E., Schulze A., Zechner R., Walther T. C., Farese R. V. (2013). Cellular fatty acid metabolism and cancer. *Cell Metabolism*.

[5] Stupp R., Taillibert S., Kanner A. A. (2015). Maintenance therapy with tumor-treating fields plus Temozolomide vs Temozolomide alone for glioblastoma: a randomized clinical trial. *JAMA*.

[6] Pandey R., Caflisch L., Lodi A., Brenner A. J., Tiziani S. (2017). Metabolomic signature of brain cancer. *Molecular Carcinogenesis*.

[7] Liu Y. Q., Chai R. C., Wang Y. Z. (2019). Amino acid metabolism‐related gene expression‐based risk signature can better predict overall survival for glioma. *Cancer Science*.

[8] Zhao S., Cai J., Li J. (2017). Bioinformatic profiling identifies a glucose-related risk signature for the malignancy of glioma and the survival of patients. *Molecular Neurobiology*.

[9] Eberlin L. S., Norton I., Dill A. L. (2011). Classifying human brain tumors by lipid imaging with mass spectrometry. *Cancer Research*.

[10] Aoki K., Nakamura H., Suzuki H. (2018). Prognostic relevance of genetic alterations in diffuse lower-grade gliomas. *Neuro-Oncology*.

[11] Ceccarelli M., Barthel F. P., Malta T. M. (2016). Molecular profiling reveals biologically discrete subsets and pathways of progression in diffuse glioma. *Cell*.

[12] Wilkerson M. D., Hayes D. N. (2010). ConsensusClusterPlus: a class discovery tool with confidence assessments and item tracking. *Bioinformatics*.

[13] Yoshihara K., Shahmoradgoli M., Martínez E. (2013). Inferring tumour purity and stromal and immune cell admixture from expression data. *Nature Communications*.

[14] Hänzelmann S., Castelo R., Guinney J. (2013). GSVA: gene set variation analysis for microarray and RNA-seq data. *BMC Bioinformatics*.

[15] Zhang C., Cheng W., Ren X. (2017). Tumor purity as an underlying key factor in glioma. *Clinical Cancer Research*.

[16] Simon N., Friedman J., Hastie T., Tibshirani R. (2011). Regularization paths for Cox's proportional hazards model via coordinate descent. *Journal of Statistical Software*.

[17] Tibshirani R., Bien J., Friedman J. (2012). Strong rules for discarding predictors in lasso‐type problems. *Series B, Statistical methodology*.

[18] Kroemer G., Pouyssegur J. (2008). Tumor cell metabolism: cancer's Achilles' heel. *Cancer Cell*.

[19] Mörén L., Bergenheim A., Ghasimi S., Brännström T., Johansson M., Antti H. (2015). Metabolomic screening of tumor tissue and serum in glioma patients reveals diagnostic and prognostic information. *Metabolites*.

[20] Dang L., White D. W., Gross S. (2009). Cancer-associated IDH1 mutations produce 2-hydroxyglutarate. *Nature*.

[21] Kallenberg K., Bock H. C., Helms G. (2009). Untreated Glioblastoma Multiforme: IncreasedMyo-inositol and Glutamine Levels in the Contralateral Cerebral Hemisphere at Proton MR Spectroscopy. *Radiology*.

[22] Nabors L. B., Portnow J., Ammirati M. (2017). NCCN guidelines insights: central nervous system cancers, version 1.2017. *Journal of the National Comprehensive Cancer Network*.

[23] Kessler T., Sahm F., Sadik A. (2018). Molecular differences in IDH wildtype glioblastoma according to MGMT promoter methylation. *Neuro-Oncology*.

[24] Salamanca-Cardona L., Shah H., Poot A. J. (2017). In Vivo Imaging of Glutamine Metabolism to the Oncometabolite 2-Hydroxyglutarate in IDH1/2 Mutant Tumors. *Cell metabolism*.

[25] Cheng W., Ren X., Zhang C. (2016). Bioinformatic profiling identifies an immune-related risk signature for glioblastoma. *Neurology*.

[26] Han S., Zhang C., Li Q. (2014). Tumour-infiltrating CD4+ and CD8+ lymphocytes as predictors of clinical outcome in glioma. *British Journal of Cancer*.

[27] Platten M., Weller M., Wick W. (2012). Shaping the glioma immune microenvironment through tryptophan metabolism. *CNS Oncology*.

[28] Picarda E., Ohaegbulam K. C., Zang X. (2016). Molecular pathways: targeting B7-H3 (CD276) for human Cancer immunotherapy. *Clinical Cancer Research*.

[29] Anderson A. C. (2014). Tim-3: an emerging target in the cancer immunotherapy landscape. *Cancer Immunology Research*.

[30] Lauer A., da X., Hansen M. B. (2017). ABCD1 dysfunction alters white matter microvascular perfusion. *Brain*.

[31] Hlavac V., Soucek P. (2015). Role of family D ATP-binding cassette transporters (ABCD) in cancer. *Biochemical Society Transactions*.

[32] Hlaváč V., Brynychová V., Václavíková R. (2013). The expression profile of ATP-binding cassette transporter genes in breast carcinoma. *Pharmacogenomics*.

[33] Bonnefont J. P., Djouadi F., Prip-Buus C., Gobin S., Munnich A., Bastin J. (2004). Carnitine palmitoyltransferases 1 and 2: biochemical, molecular and medical aspects. *Molecular Aspects of Medicine*.

[34] Fujiwara N., Nakagawa H., Enooku K. (2018). CPT2 downregulation adapts HCC to lipid-rich environment and promotes carcinogenesis via acylcarnitine accumulation in obesity. *Gut*.

[35] JO Y. S., OH H. R., KIM M. S., YOO N. J., LEE S. H. (2016). Frameshift mutations of OGDH, PPAT and PCCA genes in gastric and colorectal cancers. *Neoplasma*.

