# Supplementary material for: Bioinformatic Profiling Identifies a Fatty Acid Metabolism-Related Gene Risk Signature for Malignancy, Prognosis, and Immune Phenotype of Glioma
Source: Dis Markers. 2019 Dec 4;2019:3917040. doi: 10.1155/2019/3917040 (PMC6914924; doi:10.1155/2019/3917040)
Supplement: Supplementary Materials — Figure S1 Consensus matrixes for k = 4 to k = 10 of the 325 patients in the CGGA datasets by clustering the gene expression profile of the 73 fatty acid metabolism genes. Figure S2: Correlation of 8-gene risk signature and immune checkpoints related molecules in CGGA and TCGA datasets. Table S1: Clinicopathological features of two clusters classified by consensus clustering based on fatty acid catabolic metabolism-related gene set in CGGA dataset. Table S2: Clinicopathological features of two gene risk group in CGGA and TCGA dataset. [file 3917040.f1.docx]

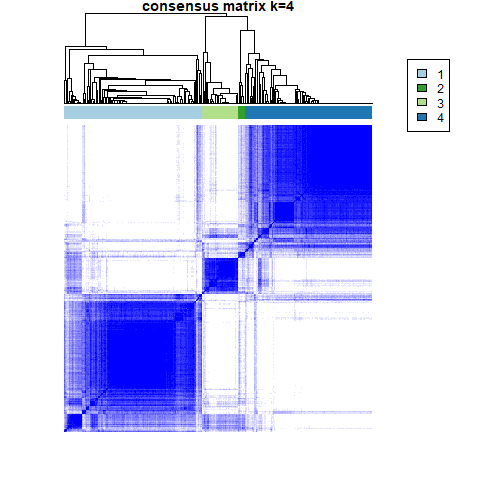

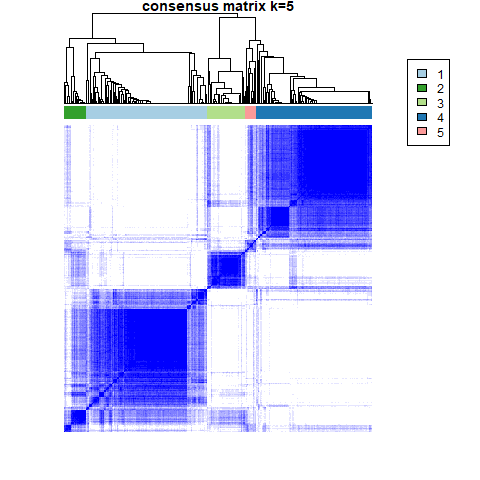


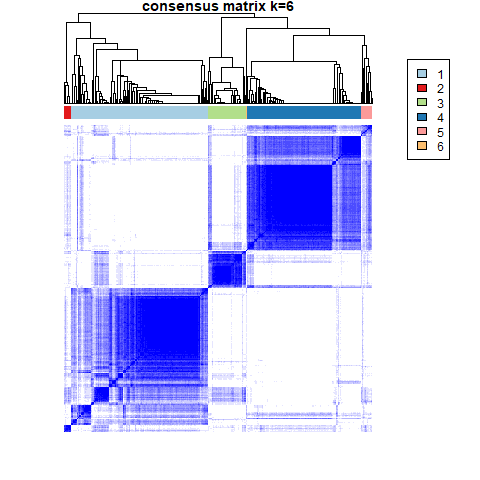

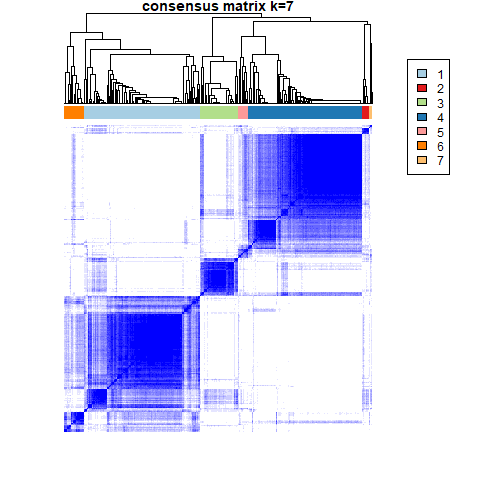


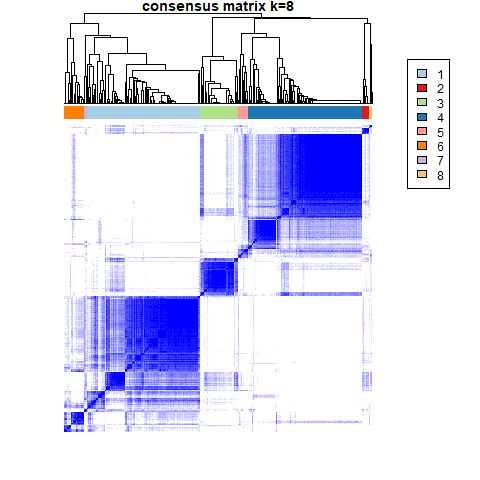

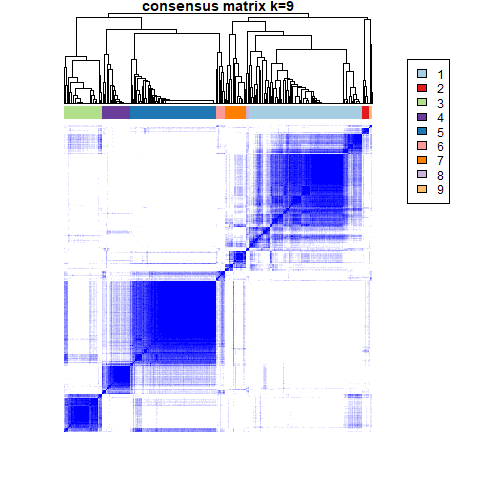


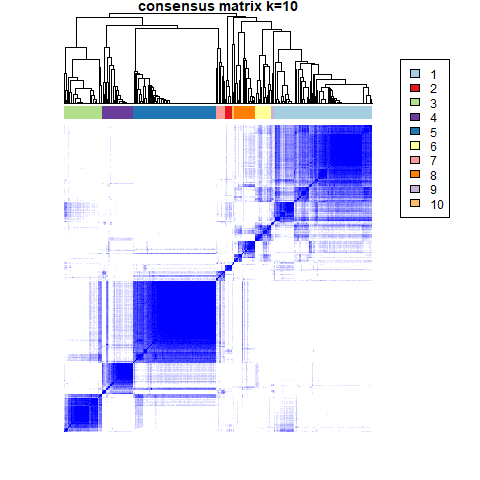


Figure S1 Consensus matrixes for k=4 to k=10 of the 325 patients in the CGGA datasets by clustering the gene expression profile of the 73 fatty acid metabolism genes.


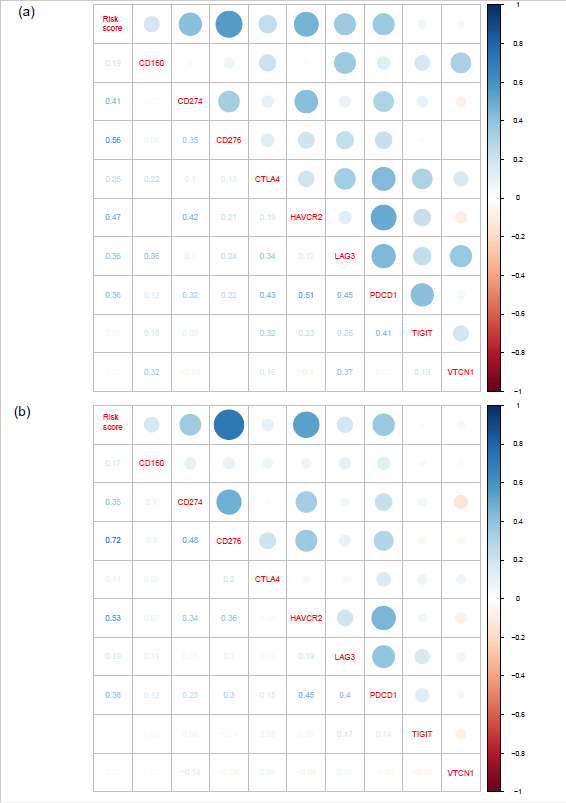


Figure S2: Correlation of 8-gene risk signature and immune checkpoints related molecules in CGGA(a) and TCGA datasets(b). The circles represent the relevance of each pair of the genes and relevance of the risk score and genes, while the numbers represent the specific R value of Pearson correlation test.

Table S1 Distribution of clinicopathological features of two clusters classified by consensus clustering based on fatty acid catabolic metabolism-related gene set in CGGA dataset.

| **Features** | **Cluster 1 (n=181)** | **Cluster 2 (n=134)** | **p-value** |
| --- | --- | --- | --- |
| Age (years) |  |  | < 0.0001 |
| Median | 45 | 39 |  |
| Gender |  |  | 0.09389 |
| Female | 64 | 58 |  |
| Male | 127 | 76 |  |
| TCGA subtype |  |  | < 0.0001 |
| Classical | 62 | 12 |  |
| Mesenchymal | 59 | 9 |  |
| Neural | 35 | 46 |  |
| Proneural | 35 | 67 |  |
| WHO grade |  |  | < 0.0001 |
| II | 30 | 79 |  |
| III | 46 | 26 |  |
| IV | 115 | 29 |  |
| IDH status |  |  | < 0.0001 |
| Mutation | 55 | 112 |  |
| Wildtype | 136 | 22 |  |
| 1p/19q status |  |  |  |
| Codeletion | 15 | 40 | < 0.0001 |
| Non-codeletion | 158 | 92 |  |
| NA | 18 | 2 |  |

Table S2 Distribution of clinicopathological features of two gene risk group in CGGA and TCGA dataset.

| **Training set CGGA RNA-seq cohort (n=325)** | | | | **Validation set TCGA RNA-seq cohort (n=667)** | | | |
| --- | --- | --- | --- | --- | --- | --- | --- |
| Features | High-risk group (n=162) | Low-risk group (n=163) | p-value | Features | High-risk group (n=333) | Low-risk group (n=334) | p-value |
| Age (years) |  |  | <0.001 | Age (years) |  |  | <0.001 |
| Median | 47.5 | 39 |  | Median | 54 | 40 |  |
| Gender |  |  | 0.448 | Gender |  |  | 0.217 |
| Female | 57 | 65 |  | Female | 132 | 150 |  |
| Male | 105 | 98 |  | Male | 199 | 184 |  |
| Subtype |  |  | <0.001 | Subtype |  |  | <0.001 |
| Classical | 57 | 17 |  | Classical | 83 | 3 |  |
| Mesenchymal | 62 | 6 |  | Mesenchymal | 93 | 4 |  |
| Neural | 8 | 73 |  | Neural | 14 | 96 |  |
| Proneural | 35 | 67 |  | Proneural | 80 | 157 |  |
| WHO grade |  |  | 0.009 | WHO grade |  |  | <0.001 |
| II | 13 | 96 |  | II | 45 | 203 |  |
| III | 35 | 37 |  | III | 132 | 130 |  |
| IV | 114 | 30 |  | IV | 155 | 1 |  |
| IDH status |  |  | <0.001 | IDH status |  |  | <0.001 |
| Mutation | 40 | 127 |  | Mutation | 116 | 309 |  |
| Wildtype | 122 | 36 |  | Wildtype | 213 | 22 |  |
| 1p/19q status |  |  | <0.001 | 1p/19q status |  |  | <0.001 |
| Codeletion | 9 | 46 |  | Codeletion | 12 | 156 |  |
| Non-codeletion | 140 | 110 |  | Non-codeletion | 314 | 178 |  |
